# Telework in health: where are we heading to?

**DOI:** 10.11606/s1518-8787.2023057004797

**Published:** 2023-05-11

**Authors:** Janete Lima de Castro, Thais Paulo Teixeira Costa, Nathalia Hanany Silva de Oliveira, Renata Rothbarth Silva, Maria Eugênia Ferraz do Amaral Bodra, Fernando Aith

**Affiliations:** I Universidade Federal do Rio Grande do Norte Departamento de Saúde Coletiva Observatório de Recursos Humanos em Saúde Natal RN Brasil Universidade Federal do Rio Grande do Norte. Departamento de Saúde Coletiva. Observatório de Recursos Humanos em Saúde. Natal, RN, Brasil; II Universidade de São Paulo. Faculdade de Saúde Pública Centro de Estudos e Pesquisas em Direito Sanitário São Paulo SP Brasil Universidade de São Paulo. Faculdade de Saúde Pública. Centro de Estudos e Pesquisas em Direito Sanitário. São Paulo, SP, Brasil

**Keywords:** Teleworking, legislation & jurisprudence, Working Conditions, Occupational Health, COVID-19

## Abstract

**OBJECTIVE:**

To identify the legal norms published in 2020 and 2021 aimed at directly or indirectly regulating telework in health in Brazil, focusing on these contexts: workday length; ergonomics; work environment; worker safety and health.

**METHODS:**

Legislative and documentary research, with a descriptive qualitative approach. We collected and selected all legal norms dealing directly or indirectly with the regulation of telework in health in Brazil, published in the bases of the National Press and Health Professional Councils until June 2021.

**RESULTS:**

Until June 2021, there were 113 valid legal norms on the regulation of telework in health, and more than half of them (64) were published in 2020. We identified only a few norms aimed at regulating or guiding aspects related to workday length; ergonomics; work environment; and worker safety and health. From the 113 norms, only one deals with workday length and just 13 pointed out the importance of working environments for offering a good service.

**DISCUSSION:**

We identified that the selected legal norms lack of devices which regulate telework in health, failing to defend workers’ and patients’ rights, or to guarantee favorable remote work conditions, whether at home or somewhere else.

## INTRODUCTION

What initiatives has the Brazilian state adopted to protect workers welfare during telework in health? What do the norms issued by professional associations and bodies of - direct and indirect - federal administration say? This study deals with these issues, contextualizing them in a society whose digital technologies seem to create illusions^1^ about a new work scenario, in which the worker would be the center of the work process.

Work conditions of health professionals in Brazil and in the world currently stand out as an important field in Public Health that should be explored and analyzed. The covid-19 pandemic brought to the center of the stage the deleterious state of work in health, especially those carried out on-site. Therefore, the precariousness of employment relationships, the lack of work environment safety, the workers’ sub conditions and vulnerability were more evident when a health crisis is added on top of several other crises the country has been facing, potentially devastating public health policies^2^.

The global pandemic also exposed that the aggravation of chronic problems that affect on-site health work conditions was followed by the emergence of new problems, typical of the digital society and expanded in the pandemic period, such as employees performing their tasks remotely through digital platforms and applications. Labor ties and relationships became, with the expansion of digital work, more flexible, unstable, intense and fragile.

Studies indicate that the employment of telework, coupled with the development of a new regulation created during the pandemic because of social distancing, will hardly return to the way it was before the pandemic^3^. A study conducted in Argentina, Brazil, Chile, Colombia and Mexico revealed that, during the Pandemic, telework increased 324% between the first and second quarter of 2020^4^. In this same direction, studies estimate that by 2050 half of the active population will work digitally^5^.

The digital legacies left by covid-19 on how we perform health work should remain even after the pandemic ends. Telework performed at home is one of these legacies^5^. In the post-pandemic scenario, it will become increasingly more difficult to distinguish work and non-work time^1^. These are some of the new and complex challenges that arise to the field of health work regulation.

This study aims at identifying the legal norms published in 2020 and 2021 focused on directly or indirectly regulating telework in health in Brazil, analyzing them under following perspectives: workday length; ergonomics; work environment; worker safety and health.

The yet scarce scientific literature on telework in health makes it currently impossible to evaluate more widely the evidence of its effects on health services and worker management. It is essential to regulate telework in health in order to fully protect not only our right to health, but also patients and professionals in this area. Thus, we highlight here the need to carefully monitor the norms that were published to regulate telework in health, so that we can make a critical analysis for their constant improvement.

It bears noticing that this article is an original analytical cutoff from the primary data and results of the research “Regulation of Telework in Health in Brazil”. This analysis had the objective of identifying and understanding how the Brazilian government is regulating remote work in health, through mapping and analyzing all legal norms (laws, decrees, ordinances and resolutions) of regulation of telework in health published in Brazil.

## METHODS

We collected and analyzed legal norms regulating health professions in Brazil in order to identify those that directly or indirectly deal with telework in health and to assess them focusing on understanding how the federal regulation is (or is not) protecting the workers’ and patients’ rights. In this sense, we identified and analyzed the legal norms and the specific devices in each of them that regulate the following relevant issues of Public Health and occupational health in the context of telework: workday length; ergonomics; work environment; safety and health of the worker.

The concept of Health Profession used here refers to resolution n. 287, of October 8, 1998, of the National Council of Health^7^, which encompasses 14 higher-level health professions working at the council. For these 14 professions, we mapped 13 legally competent professional councils publishing legal norms for the work regulation in each of these professions, and the Federal Council of Physiotherapy and Occupational Therapy is responsible for these two professions.

We designed a data collection form with the purpose of organizing and selecting general information about each normative act, such as publication date, the name and the issuing body, specific information regarding working conditions and information security and personal data protection. For the Bills, a specific form was prepared, highlighting the central information of the document, authorship, legislative chamber, publication dates, syllabus and whether or not the PL repealed the existing norm.

We hosted the form on a platform at the Universidade Federal do Rio Grande do Norte (UFRN), allowing the research team safe and functional access to the instrument and feeding the database with the strategic information of the normative documents.

Data collection on legal norms took place between February, March, April, May and June 2021, with June 30, 2021 being the research time frame. In this stage, we collected normative texts, as a priority, directly from the Official Diary of the Union. After this, we searched other websites of regulatory institutions. While we read and collected this data set, we continuously filled in the aforementioned form.

Before the collection, we defined the modalities of legal norms we were interested in, which were: constitutional provisions, complementary laws, ordinary laws, presidential decrees, legislative decrees, delegated laws, provisional measures, ordinances, resolutions, normative resolutions, normative instructions, decisions, circulaires, communications and opinions. We previously excluded all legal norms relating to disciplinary, sanctioning and contracting administrative processes, for example, bidding.

We selected the official base of the National Press^8^ and the bases made available by Professional Councils. At first, the search strategy defined the following descriptors: teleconsultation, telehealth, teleworking, teleassistance, *teleprofession* (e.g. telemedicine), digital health, distance consultation, distance care, remote (masculine and feminine), *home office*, informatics, information and communication technologies.

The search in the National Press database was carried out in the “Act-by-Act search” mode, which selects all the retrieved legal norms containing the indicated descriptor. We filtered the search according to institution or public body issuing the norm. The Table below presents all public agencies and institutions issuing legal norms that regulate teleworking in Brazil consulted by the search through reading the National Press:

**Table:** Public agents issuing legal norms consulted by the research. Brazil. 2021.

DIRECT PUBLIC ADMINISTRATION (FEDERAL)	INDIRECT PUBLIC ADMINISTRATION (Regulatory Agencies)	INDIRECT PUBLIC ADMINISTRATION (Health Professional Councils, including Regional Councils)
National Congress	National Health Surveillance Agency	Federal Council of Medicine
Presidency of the Republic	National Supplementary Health Agency	Federal Council of Biomedicine
Ministry of Health	National Data Protection Authority	Federal Council of Biology
Ministry of Education		Federal Nursing Council
Ministry of Economy		Federal Council of Psychology
Ministry of Labor and Employment		Federal Council of Dentistry
		Federal Council of Pharmacy
		Federal Council for Nutrition
		Federal Council of Physical Education
		Federal Council of Social Work
		Federal Council of Veterinary Medicine
		Federal Council of Speech Therapy
		Federal Council of Physiotherapy and Occupational Therapy

Source: Regulation of Telework in Health in Brazil Research Report, 2021^7^.

The National Press database allows the search of legal norms published between the search date (from February to June 2021) and 2018. This database gives access to all the Official Diary of the Union publications. The search focused on Section 1 of the Official Diary of the Union, composed by normative acts (legal norms), excluding Section 2, composed by personnel acts and Section 3, composed by contracts, announcements and notices. We selected the legal norms published between January 2018 and June 30, 2021 regarding the Union. In the first analysis, we read these documents to identify direct references on norms prior to 2018 and, thus, to add these complementary norms to the set of collected norms.

After that, the documentary bases of the Professional Councils (both federal and regional) and public administration entities were sought, in an exploratory manner. This stage did not consider a time frame, since we carried out a floating reading in the documents published in each repository, based on the set of descriptors chosen for this research, so that we could understand discussions and current institutional frameworks on the topic.

Finally, we also included, for all regulated professions, the location and analysis of each ethics code. Although most of these codes lack of the indicated descriptors, such norms often deal with treatment confidentiality and other relevant topics for understanding the regulation of distance therapy.

Hence, we obtained all legal norms according to the aforementioned descriptors, including those repealed between January 2018 and June 2021, as well as all the legal norms in force that regulate teleworking in health.

As a result of the research, 113 legal norms of the Union were selected for analysis.

## RESULTS AND DISCUSSION

The health sector in Brazil has always been the stage for regulatory conflicts that cover various topics, such as: the definition of the practice scopes of each profession; the definition of training requirements to work in certain activities; working hours; and the compensation for each different health professional^10^. These are some of the old and current challenges faced by those who manage labor in public health institutions. Recently, with the growth of telework in health, new challenges related to its regulation have emerged^10^_._

A research published by the Institute for Applied Economic Research (Ipea) reveals that the amount of overall telework in Brazil is already quite significant when compared to other countries around the world, showing that, in a list of 86 countries ranked according to the proportion of telework use, Brazil places at the 45th position^11^. In the health sector, this type of work has been increasingly present, with its expansion increasing during the covid-19 pandemic. However, it is important to emphasize that “the conversion of on-site work into a remote one, in the pandemic context, consisted of a health contingency”^11^.

Once this moment is over, the maintenance or reversal of remote work relationships should be well evaluated from the perspective of not increasing the precariousness of health work and intensifying the workers exploitation in the direction of digital servitude, as reflected by Antunes^12^. It is also important to ensure that teleworking in health does not compromise the quality and efficiency of the provided service, protecting patients from potential risks involved in this new way of providing service.

The results of this research revealed that until June 30, 2021, Brazil had 113 legal norms in force regulating telework in health, and more than half of them (64 norms) were issued in 2020. In 2021, eight new published norms were identified – however, as part of the survey collection finished in March 2021, it is very likely that the number of norms published throughout 2021 on the topic should be higher than eight.

Corroborating the data from the Ipea survey regarding overall telework, this study demonstrates that the topic had already been gaining importance in recent years, even before the pandemic. For instance, in 2018 and 2019 nine and 11 norms were published respectively, which is twice as much as what was previously published, as shown in the Figure.

**Figure:**
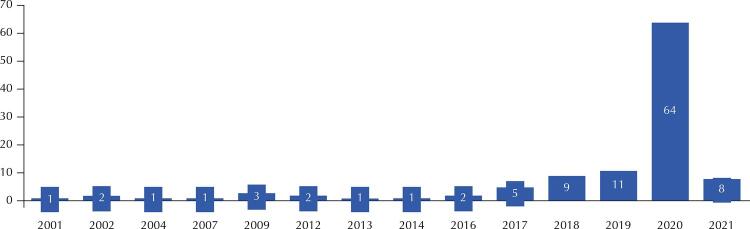
Legal norms that regulate teleworking in health by year of issue.

Source: Regulation of Telework in Health in Brazil Research Report, 2021^7^. Data for 2021 are limited to the period from January to June 2021.

The legal norms that today regulate telework in Brazil were issued by different federal agencies and institutions. The federal and regional Professional Councils were responsible for publishing 97 norms regulating telework in health, divided as follows: Pharmacy Councils (2); Speech Therapy Councils (9); Psychology Councils (23); Nursing Councils (1); Medicine Councils (30); Nutrition Councils (4); Physical Education Councils (8); Dentistry Councils (8); Physiotherapy and Occupational Therapy Councils (3); Biomedicine Councils (1); Social Work Councils (7) and an Ordinance on the joint between Regional Councils of Psychology, Social Work and Physiotherapy and Occupational Therapy of the State of Minas Gerais (1).

The remaining 16 legal norms were published by the Presidency of the Republic (laws and decrees), the Ministry of Health, the Ministry of Labor and Employment, the Ministry of Economy and the National Supplementary Health Agency (ANS)^9^

When directing efforts to analyze the content of such norms, in the context of telework in health, it is observed that this field is little explored by the current regulation. The search for this type of content in the selected legal norms found that out of the 113 norms, only one deals with the workday length and another one has a device regulating the control of the daily teleworking hours; only one norm contains a device on ergonomics; seven norms have devices about worker safety and health, 12 norms regarding the guarantee of care quality; and 13 norms on the work environment.

The in-depth analysis of these norms that regulate working hours, ergonomics and work environment brings important data. With regard to working hours, the only normative act found in the studied period was normative Instruction No. 65, of July 30, 2020, published by the Ministry of Economy, which presents the guidelines, criteria and general procedures to be observed by the bodies and entities that are part of the Civilian Personnel System of the Federal Administration (Sipec) regarding the implementation of a management program. This instruction states that, when the telework model to which the participant is subjected to comprises their entire workday, they are exempted from attendance control. This normative act says nothing about the length or workday time control.

In the specific legal norm that deals with the time control of the working day (Order of March 26, 2018, from the Ministry of Labor and Employment), the text highlights that employees in a teleworking regime are not covered by the working day format, being excluded from this protection, as well as from the other rights arising from Title II of the Consolidation of Labor Laws (CLT), such as, night shift extra, overtime or any other compensation that is earned through controling the workday length. It should be noted that since the Labor Reform of 2017, remote work is no longer bound by the control of working hours, nor by the payment of overtime^13^.

Regarding the normative content that regulates ergonomics and the work environment, thirteen norms highlighted the importance of work environments meeting adequate conditions to offer a good service. They are: 1. Resolution No. 666 of September 30, 2020, issued by the Federal Nutrition Council^14^; 2. Recommendation of the Regional Council for Social Work of the 1st Region No. 01/2020 of March 20, 2020^15^; 3. Resolution of the Regional Council of Social Service of Sergipe (CRESS/SE) No. 01 of March 25, 2020^16^; 4. Technical note No. 01/2020, issued by the Regional Council of Social Service of Acre^17^; 5. Resolution No. 007/2020 issued by the Regional Council of Dentistry of Minas Gerais^18^; 6. Resolution No. 02/2019, of January 16, 2019, issued by the Regional Council of Psychology – Federal District^19^; 7. Protocol of the Regional Council of Psychology for the “Coexistence Plan of the State Office of Health of Pernambuco to face the Coronavirus Pandemic –covid 19”, published in June 2020^20^; 8. Resolution of the Regional Council of Psychology 15, No. 003, of November 28, 2019^21^; 9. Resolution of the Regional Council of Psychology -18 / MT No. 002/2019 of January 30, 2018^22^; 10. Resolution No. 01, of January 11, 2019,issued by the Federal Council of Psychology^23^; 11. Normative Instruction No. 65 ofJuly 30, 2020, issued by the Ministry of Economy^24^; 12. Resolution of the Federal Council of Medicine No. 2.235/2019 of October 1, 2019^25^; 13. Resolution of the Regional Council of Medicine of the State of Bahia No. 367/2020^26^.

Although such norms present devices that regulate ergonomics and the working environment, they say little about the employer’s duties to ensure that this occurs.Sometimes the meaning is even reversed, for example, the content about working conditions contained in Article 23 of the Normative Instruction of the Ministry of Economy No. 65 of July 30, 2020^24^:

When teleworking, it will be the responsibility of the participant to provide the necessary physical and technological structures, through the use of appropriate and ergonomic equipment and furniture, including the costs related to internet connection, electricity and telephone, among other expenses arising from the performance of their duties.

Although in Brazil there is little concern about the risks arising from teleworking, it is necessary to remember that, if there is a process of implementing it, the employer should consider all strategies that can reduce or control occupational risk factors that can affect workers’ health^27^. In this sense, in Colombia, for example, the ABC of Teleworking about the management of occupational risks in teleworking presents a checklist that includes, among other aspects “the verification of jobs, in addition to proposing and implementing intervention measures associated with the workplace and raising awareness of self-reporting of working conditions”^28^ (P. 96).

Among the advices and recommendations given for the implementation of teleworking,in the aforementioned document, are:

affiliation to the Social Security System, knowing the teleworker’s workplace conditions, ensuring that the teleworker self-reports their working conditions, adding in the internal Labor Regulations, the teleworking contracting model, defining the statutory hours of work per day and per week, making the necessary corrections to work environments, training processes, trainings, among others^28^ (P. 99).

On the other hand, the agreement on teleworking between social agents in the European Union, in 2002, “points out the difficulty of regulating this new form of work organization and the Prevention of possible injuries and associated diseases, especially mental illnesses and disorders”^6^ (P. 1).

The results found here attest to the rapid and exponential growth of telework in health in Brazil, reflected in the normative regulation issued by different bodies and regulatory institutions in the country. If even before the pandemic the use and regulation of remote work were timid and advanced gradually, the pandemic tried to accelerate this process of institutionalization of telework in health, in a seemingly irreversible way. It should be noted, in this sense, the large number of legal norms published in the years 2020 and 2021^9^, the heaviest years of the covid-19 pandemic, which remain in force even after the end of the health emergency.

However, at the same time that the usage and regulation of this practice increased, there is no adequate protection for either the health professional or the patient in the published state regulation. Relevant topics such as the workday length, working time control, ergonomics and the working environment were completely ignored or insufficiently addressed, leaving workers and patients in a vulnerable situation.

The understanding of this phenomenon cannot be dissociated from the movements of the world of work in general, about which the literature^29^ has long presented critical reflections on the precarious working conditions and their deleterious effects on workers’ health, for example, Burnout syndrome, a type of occupational stress that affects professionals involved in any type of healthcare in a relationship of direct, continuous and highly emotional attention^31^.

A survey carried out on the ergonomic conditions in which workers from various economic sectors in Brazil who performed remote work in the first year of the covid-19 pandemic identified that a large part of them evaluates these conditions as “reasonable”, because they have some technological equipment, or “terrible”, because they do not have space and conditions to work remotely.^32^_._ We observed that the evaluations were concentrated between reasonable and terrible, which makes evident the need to monitor the implementation of telework in health in Brazil and to give special attention to the publishing of negligent or even deleterious legal norms regarding the protection of teleworker rights in the health area in Brazil.

## FINAL CONSIDERATIONS

When assessing the selected material, we identified a lack of norms issued by health work regulatory agencies and institutions in Brazil that can defend decent working conditions in teleworking in health. Moreover, they are insufficient to induce employers to create favorable conditions for teleworking, whether at home or in another private space. This is a necessary, relevant and complex discussion that involves fundamental rights, such as the confidentiality and privacy of personal data; ethical duties; the responsibilities of health professionals; health protection; and the working conditions of the employee and the service provider. Therefore, health workers and managers must remain vigilant and act quickly to protect their work, employment, service and, above all, the user/patient.

Studying teleworking and its effects on work, health services provision and worker integrity should be an urgent task for those who work with management and regulation of health work. Including this topic in the manager’s agenda may minimize the possible deleterious effects on health work management and improve the content and respective applications of legal regulations. We draw attention to the importance of the worker’s health and safety as one of the central elements in this discussion.

There is much to learn about this new work modality, because, according to the results presented here, it is possible that the reality of teleworking will grow after the pandemic. Thus, we reinforce that vigilance is necessary, so that this new modality of health work and its technologies do not intensify existing inequalities in the labor world, nor contribute to the creation of hazardous working environments.

This study shows a normative gap to be understood and overcome, as well as exposes the still primary, fragmented and hesitant treatment of the regulation of teleworking in health in its various aspects in Brazil. In this regard, we draw attention to the importance of improving teleworking regulation, so that it, contrary to the current scenario, is effectively protective towards the patient’s right to health and the worker’s rights of health professionals.

Considering that teleworking is already present in the public and private health systems, its regulatory agenda is an urgent and current challenge since it is already part of the daily life of health institutions.
