# Supplementary material for: Telework in health: where are we heading to?
Source: Rev Saude Publica. 2023 May 11;57(Suppl 1):7s. doi: 10.11606/s1518-8787.2023057004797 (PMC10185311; doi:10.11606/s1518-8787.2023057004797)
Supplement: Translation

# Translation: Teletrabalho em saúde: para onde vamos?

**Authors:** Janete Lima de Castro, Thais Paulo Teixeira Costa, Nathalia Hanany Silva de Oliveira, Renata Rothbarth Silva, Maria Eugênia Ferraz do Amaral Bodra, Fernando Aith

**Affiliations:** I Brasil Universidade Federal do Rio Grande do Norte. Departamento de Saúde Coletiva. Observatório de Recursos Humanos em Saúde. Natal, RN, Brasil; II Brasil Universidade de São Paulo. Faculdade de Saúde Pública. Centro de Estudos e Pesquisas em Direito Sanitário. São Paulo, SP, Brasil

**Keywords:** Teletrabalho, legislação e jurisprudência, Condições de Trabalho, Saúde do Trabalhador, COVID-19

## Abstract

**OBJETIVO:**

Identificar as normas jurídicas publicadas nos anos de 2020 e 2021 para regular direta ou indiretamente o teletrabalho em saúde no Brasil, analisando-as sob o enfoque dos seguintes temas, no contexto do teletrabalho: tempo da jornada de trabalho; ergonomia; ambiente laboral; segurança e saúde do trabalhador.

**MÉTODOS:**

Pesquisa legislativa e documental, com abordagem qualitativa descritiva. Foram coletadas e selecionadas todas as normas jurídicas que tratam direta ou indiretamente da regulação do teletrabalho em saúde no Brasil, publicadas nas bases da Imprensa Nacional e dos Conselhos Profissionais da área da saúde até junho de 2021.

**RESULTADOS:**

Destacam-se os seguintes: até junho de 2021 havia 113 normas jurídicas vigentes sobre a regulação do teletrabalho em saúde, sendo mais da metade (64) publicadas no ano de 2020. Dentre os achados, identificou-se um baixo número de normas voltadas a regular ou orientar aspectos relacionados ao tempo da jornada de trabalho; à ergonomia; ao ambiente laboral; à segurança e à saúde do trabalhador. Do total das 113 normas, apenas uma trata da jornada de trabalho e somente 13 destacaram a importância de que os ambientes de trabalho tenham condições para o oferecimento de um bom serviço.

**DISCUSSÃO:**

Identificamos ausências, nas normas jurídicas selecionadas, de dispositivos que regulam o teletrabalho em saúde, a fim de prover a defesa dos direitos do trabalhador e dos pacientes, ou ainda para garantir condições favoráveis à realização do teletrabalho, seja realizado nos domicílios, seja em outro espaço.

## INTRODUÇÃO

Quais têm sido as iniciativas adotadas pelo Estado brasileiro para a proteção da saúde do trabalhador durante o exercício do teletrabalho em saúde? O que dizem as normas emitidas pelas entidades profissionais e pelos órgãos da administração - direta e indireta - federal? Este trabalho versa acerca dessas questões, contextualizando-as numa sociedade cujas tecnologias digitais parecem criar ilusões^1^ sobre um novo mundo do trabalho, no qual o trabalhador seria o centro do processo de trabalho.

As condições do exercício profissional da força de trabalho em saúde hoje no Brasil e no mundo representam um campo importante da saúde pública que deve ser mais bem conhecido e analisado. A pandemia causada pelo coronavírus trouxe ao centro do palco as condições deletérias do trabalho em saúde, sobretudo, aqueles trabalhos que foram realizados de forma presencial. Assim, a precarização dos vínculos empregatícios, a falta de proteção no ambiente de trabalho, as subcondições e a vulnerabilidade do trabalhador ficaram mais evidentes em um contexto cuja crise sanitária se soma às múltiplas crises que o país vem enfrentando, com efeitos potencialmente devastadores sobre as políticas públicas de saúde^2^.

A pandemia da covid-19 também deixou evidente que o aprofundamento dos problemas crônicos que impactam as condições do trabalho em saúde desenvolvidos presencialmente foi acompanhado pelo surgimento de novos problemas, próprios da sociedade digital e ampliados no período pandêmico, por exemplo, o exercício do trabalho domiciliar pelos empregados, que passam a realizar suas tarefas sob demandas virtuais, mediante plataformas e aplicativos digitais. Os vínculos e relações trabalhistas tornaram-se, com a ampliação do trabalho digital, mais flexíveis, instáveis, intensos e frágeis.

A literatura sinaliza que a utilização do teletrabalho, aliado ao surgimento de uma nova regulação incrementada durante a pandemia como consequência do distanciamento social, dificilmente retornará à forma como era antes da pandemia^3^. Estudo realizado na Argentina, Brasil, Chile, Colômbia e México revelou que, durante a pandemia, houve um aumento de 324%, no teletrabalho, entre o primeiro e o segundo trimestre de 2020^4^. Nessa mesma direção, estima-se que até 2050 metade da população ativa realizará seu trabalho por meios digitais^5^.

As heranças digitais deixadas pela covid-19 na forma de realizar trabalho em saúde deverão permanecer mesmo após superada a pandemia. O teletrabalho executado no domicílio se apresenta como uma dessas heranças^5^. No cenário pós-pandêmico ficará cada vez mais difícil distinguir o tempo do trabalho e do não trabalho^1^. Esses são alguns dos novos e complexos desafios que se apresentam para o campo da regulação do trabalho em saúde.

Este artigo pretende identificar as normas jurídicas publicadas nos anos de 2020 e 2021 para regular direta ou indiretamente o teletrabalho em saúde no Brasil, analisando-as sob o enfoque dos seguintes temas, no contexto do teletrabalho: tempo da jornada de trabalho; ergonomia; ambiente laboral; segurança e saúde do trabalhador.

A ainda escassa literatura científica sobre teletrabalho em saúde impossibilita avaliar com maior amplitude, nesse momento, as evidências dos seus efeitos nos serviços de saúde e na gestão do trabalhador. É fundamental que a regulação do teletrabalho em saúde seja realizada para a plena proteção do direito à saúde, englobando proteção aos pacientes e aos profissionais da área. Assim, o que se coloca em perspectiva neste artigo é a necessidade de se acompanhar com atenção as normas que vêm sendo editadas para regular o teletrabalho em saúde, possibilitando uma análise crítica para o seu aperfeiçoamento constante.

Cumpre frisar que este artigo é um recorte analítico original dos dados primários e resultado da pesquisa “Regulação do Teletrabalho em Saúde no Brasil”, cujo objetivo consistiu em identificar e compreender como o teletrabalho em saúde está sendo regulado no Brasil, por meio do mapeamento e análise de todas as normas jurídicas (leis, decretos, portarias e resoluções) de regulação do teletrabalho em saúde editadas no Brasil.

## MÉTODOS

Foram coletadas e analisadas as normas jurídicas que regulam as profissões de saúde no Brasil, para fins de identificar aquelas que tratam direta ou indiretamente do teletrabalho em saúde e analisá-las com enfoque para a compreensão sobre de que forma a regulação estatal do teletrabalho em saúde no Brasil está (ou não está) protegendo os direitos do trabalhador e dos pacientes. Nesse sentido, foram identificadas e analisadas as normas jurídicas e os dispositivos específicos de cada uma delas que tratavam de regular os seguintes temas de saúde pública e saúde do trabalhador, relevantes no contexto do teletrabalho: tempo da jornada de trabalho; ergonomia; ambiente laboral; segurança e saúde do trabalhador.

O conceito de profissão de saúde utilizado tem como referência a Resolução n. 287, de 8 de outubro de 1998, do Conselho Nacional de Saúde^7^, que relaciona 14 profissões de saúde de nível superior para os fins da atuação do conselho. Para essas 14 profissões foram mapeados 13 conselhos profissionais com competência legal de publicar normas jurídicas que para a regulação do exercício profissional para cada uma dessas profissões, sendo que o Conselho Federal de Fisioterapia e Terapia Ocupacional apresenta competência regulatória sobre essas duas profissões.

O formulário que orientou a coleta de dados para a pesquisa foi elaborado com o fim de organizar e selecionar informações gerais acerca de cada ato normativo encontrado, por exemplo, a data de publicação, o nome e o órgão emissor, informações específicas quanto às condições de trabalho e segurança da informação e proteção de dados pessoais. Para os projetos de lei, foi elaborado formulário específico, destacando informações centrais do documento, autoria, casa legislativa, data de publicação, ementas e se o PL revogava ou não a norma existente.

O formulário foi hospedado em uma plataforma da Universidade Federal do Rio Grande do Norte (UFRN) desenvolvida para esse fim, permitindo à equipe de pesquisa acesso seguro e funcional ao instrumento e a alimentação da base de dados criada com as informações estratégicas dos documentos normativos encontrados.

A coleta de informações associadas às normas jurídicas aconteceu entre os meses de fevereiro, março, abril, maio e junho de 2021, sendo 30 de junho de 2021 o recorte temporal da pesquisa. Nessa etapa metodológica a coleta dos textos normativos foi feita, de modo prioritário, diretamente nos diários oficiais da União. Em seguida, foram pesquisados outros *sites* das instituições reguladoras. Ao longo da leitura e da coleta desse conjunto de dados, o formulário foi preenchido de forma contínua.

Antes da coleta, foram definidas as modalidades de normas jurídicas de interesse para a pesquisa, sendo eles: dispositivos constitucionais, leis complementares, leis ordinárias, decretos presidenciais, decretos legislativos, leis delegadas, medidas provisórias, portarias, resoluções, resoluções normativas, instruções normativas, decisões, circulares, comunicados e pareceres. Foram previamente excluídas todas as normas jurídicas relativas aos processos administrativos disciplinares, sancionatórios e de contratação, por exemplo, licitação.

As bases oficiais selecionadas foram a da Imprensa Nacional^8^ e as bases disponibilizadas pelos próprios Conselhos Profissionais. A princípio, a estratégia de busca definiu os seguintes descritores: teleconsulta, telessaúde, teletrabalho, teleassistência, *teleprofissão* (por exemplo, telemedicina), saúde digital, consulta à distância, atendimento à distância, remoto, remota, *home office*, informática, tecnologias da informação e comunicação

A busca na base da Imprensa Nacional foi feita na modalidade “Pesquisa Ato a Ato”, que seleciona todas as normas jurídicas encontradas que contém o descritor indicado. A busca foi filtrada conforme a instituição ou órgão público emissor da norma. O Quadro, a seguir, apresenta todos os órgãos públicos e instituições emissoras de normas jurídicas que regulam o teletrabalho no Brasil consultados pela pesquisa por meio da leitura da Imprensa Nacional:

**Quadro:** Agentes públicos emissores de normas jurídicas consultados pela pesquisa. Brasil. 2021.

ADMINISTRAÇÃO PÚBLICA DIRETA (FEDERAL)	ADMINISTRAÇÃO PÚBLICA INDIRETA (Agências Reguladoras)	ADMINISTRAÇÃO PÚBLICA INDIRETA (Conselhos Profissionais de Saúde, incluindo Conselhos Regionais)
Congresso Nacional	Agência Nacional de Vigilância Sanitária	Conselho Federal de Medicina
Presidência da República	Agência Nacional de Saúde Suplementar	Conselho Federal de Biomedicina
Ministério da Saúde	Autoridade Nacional de Proteção de Dados	Conselho Federal de Biologia
Ministério da Educação		Conselho Federal de Enfermagem
Ministério da Economia		Conselho Federal de Psicologia
Ministério do Trabalho e Emprego		Conselho Federal de Odontologia
		Conselho Federal de Farmácia
		Conselho Federal de Nutrição
		Conselho Federal de Educação Física
		Conselho Federal de Serviço Social
		Conselho Federal de Medicina Veterinária
		Conselho Federal de Fonoaudiologia
		Conselho Federal de Fisioterapia e Terapia Ocupacional

Fonte: Relatório da Pesquisa Regulação do Teletrabalho em Saúde no Brasil, 2021^7^.

A base de dados da Imprensa Nacional permite a busca de normas jurídicas editadas entre a data da busca (de fevereiro a junho de 2021) e o ano de 2018. Essa base dá acesso ao conjunto de todas as publicações do Diário Oficial da União. A busca centrou-se na Seção 1 do Diário Oficial da União, formada por atos normativos (normas jurídicas), excluindo-se a Seção 2, formada por atos de pessoal e a Seção 3, formada por contratos, editais e avisos. Foram inicialmente selecionadas as normas jurídicas publicadas entre janeiro de 2018 e 30 de junho de 2021 no âmbito da União. Esses documentos foram lidos, em primeira análise, a fim de identificar referências diretas sobre normas anteriores a 2018 e, assim, agregar essas normas complementares ao conjunto de normas coletadas.

Em seguida, buscou-se as bases documentais dos próprios Conselhos Profissionais (federais e regionais) e entidades da administração pública, de maneira exploratória. Essa etapa não considerou um recorte temporal, uma vez que foi realizada uma leitura flutuante nos documentos publicados em cada repositório, tendo como base o conjunto dos descritores escolhidos para esta pesquisa, pelo interesse em compreender discussões e marcos institucionais vigentes acerca do tema.

Por fim, incluiu-se também, para todas as profissões reguladas, a localização e análise de cada Código de Ética. Ainda que boa parte desses Códigos não contenham os descritores indicados, tais normas frequentemente versam sobre confidencialidade do tratamento e outros temas relevantes para a compreensão da regulação da terapêutica à distância.

Desse modo, obteve-se assim todas as normas jurídicas, inclusive as revogadas, entre janeiro de 2018 e junho de 2021, bem como todas as normas jurídicas em vigor que regulam o teletrabalho em saúde, obtidos de acordo com os descritores mencionados.

Como resultado da pesquisa, foram selecionadas para análise, no total, 113 normas jurídicas da União.

## RESULTADOS E DISCUSSÃO

O setor de Saúde no Brasil tem sido palco de conflitos regulatórios que abrangem temas diversos, tais como: a definição dos escopos de prática de cada profissão; a definição das exigências de formação para o exercício de determinadas atividades; as jornadas de trabalho; e as remunerações a serem percebidas pelos diferentes profissionais de saúde^10^. Esses são alguns dos desafios antigos e atuais enfrentados por aqueles que fazem a gestão do trabalho nas instituições públicas de saúde. Recentemente, com o crescimento do teletrabalho na área da saúde, novos desafios relacionados à regulação do exercício do teletrabalho das profissões de saúde surgiram^10^_._

Pesquisa publicada pelo Instituto de Pesquisa Econômica Aplicada (Ipea) revela que o uso do teletrabalho em geral no Brasil já é bastante significativo quando comparado a outros países ao redor do mundo, mostrando que, em uma lista de 86 países classificados em relação à proporção de uso do teletrabalho, o Brasil ocupa a 45ª posição^11^. No setor de saúde, essa modalidade de trabalho vem se fazendo cada vez mais presente, tendo sua expansão incrementada no período da pandemia covid-19. Contudo, é importante frisar que “a conversão das formas de trabalho presenciais em remotas, no contexto pandêmico, consistiu em contingência sanitária”^11^.

Superado esse momento, a manutenção ou reversão das relações de trabalho remoto deverá ser bem avaliada na perspectiva de não se ampliar a precarização do trabalho em saúde e intensificar a exploração dos trabalhadores no sentido da servidão digital, conforme reflete Antunes^12^. Também deve-se cuidar para que o teletrabalho em saúde não comprometa a qualidade e eficiência do serviço prestado, protegendo os pacientes dos potenciais riscos que envolvem essa nova forma de prestar serviço.

Os resultados desta pesquisa revelaram que até 30 de junho de 2021, o Brasil tinha 113 normas jurídicas vigentes regulando o teletrabalho em saúde, sendo que mais da metade delas (64 normas) foram emitidas no ano de 2020. No ano de 2021 foram identificadas oito novas normas publicadas – no entanto, como parte da coleta da pesquisa foi encerrada em março de 2021, é muito provável que o número de normas editadas durante todo o ano de 2021 sobre o tema seja maior do que oito.

Corroborando os dados da pesquisa do Ipea em relação ao teletrabalho geral, a pesquisa ora apresentada demonstra que o tema já vinha ganhando importância nos últimos anos, mesmo antes da pandemia, já que em 2018 e 2019 foram editadas nove e 11 normas respectivamente, número que é maior do que o dobro do que foi editado em todos os anos anteriores, conforme demonstrado na Figura.

**Figura:** Normas jurídicas que regulam o teletrabalho em saúde por ano de edição.

Fonte: Relatório da Pesquisa Regulação do Teletrabalho em Saúde no Brasil, 2021^7^. Os dados de 2021 limitam-se ao período de janeiro a junho de 2021.

As normas jurídicas que hoje regulam o teletrabalho no Brasil foram emitidas por diferentes órgãos e instituições federais. Os Conselhos Profissionais Federais e Regionais foram responsáveis pela edição de 97 normas regulando teletrabalho em saúde, divididas da seguinte forma: Conselhos de Farmácia (2); Conselhos de Fonoaudiologia (9); Conselhos de Psicologia (23); Conselhos de Enfermagem (1); Conselhos de Medicina (30); Conselhos de Nutrição (4); Conselhos de Educação Física (8); Conselhos de Odontologia (8); Conselhos de Fisioterapia e Terapia Ocupacional (3); Conselhos de Biomedicina (1); Conselhos de Serviço Social (7) e uma Portaria em conjunto entre Conselhos Regionais de Psicologia, Serviço Social e Fisioterapia e Terapia Ocupacional do Estado de Minas Gerais (1).

As 16 normas jurídicas restantes foram publicadas pela Presidência da República (Leis e Decretos), pelo Ministério da Saúde, pelo Ministério do Trabalho e Emprego, pelo Ministério da Economia e pela Agência Nacional de Saúde Suplementar (ANS)^9^

Ao direcionar esforços para análise de conteúdo de tais normas no tema das condições de trabalho, no contexto do teletrabalho em saúde, observa-se que esse tema constitui um campo pouco explorado pela regulação atual. A busca por esse tipo de conteúdo nas normas jurídicas selecionadas constatou que das 113 normas, apenas uma norma trata da jornada de trabalho e uma outra apresenta dispositivo regulando o controle do tempo da jornada de teletrabalho; somente uma norma contém dispositivo sobre ergonomia; sete normas apresentam dispositivos acerca da segurança e saúde do trabalhador, 12 normas quanto à garantia da qualidade do cuidado; e 13 normas sobre ambiente de trabalho.

A análise aprofundada dessas normas que regulam jornada de trabalho, ergonomia e ambiente do trabalho traz dados importantes. No que diz respeito à jornada de trabalho, o único ato normativo encontrado no período estudado foi a Instrução Normativa nº 65, de 30 de julho de 2020, publicada pelo Ministério da Economia, que apresenta as orientações, critérios e procedimentos gerais a serem observados pelos órgãos e entidades integrantes do Sistema de Pessoal Civil da Administração Federal (Sipec) relativos à implementação de Programa de Gestão. Essa instrução afirma que, quando a forma de teletrabalho a que está submetido o participante compreende a totalidade da sua jornada de trabalho, ele é dispensado do controle de frequência. Esse ato normativo nada diz em relação ao tempo ou controle de tempo da jornada.

Na norma jurídica específica que trata do controle de tempo da jornada de trabalho (Despacho de 26 de março de 2018, do Ministério do Trabalho e Emprego), o texto da norma destaca que os empregados em regime de teletrabalho não estão abrangidos pelo formato de jornada de trabalho, estando excluídos dessa proteção, bem como dos demais direitos provenientes do Título II da Consolidação das Leis Do Trabalho (CLT), tais como, adicional noturno, horas extras ou qualquer outro que seja auferido por meio de controle da jornada de trabalho. Ressalta-se que desde a Reforma Trabalhista de 2017, o trabalho remoto não está mais sujeito ao controle de jornada, tampouco ao pagamento de horas extras^13^.

Quanto ao conteúdo normativo que regula ergonomia e ambiente de trabalho, treze normas destacaram a importância de que os ambientes de trabalho atendam às condições para o oferecimento de um bom serviço. São elas: 1. Resolução nº 666, de 30 de setembro de 2020, emitido pelo Conselho Federal de Nutrição^14^; 2. Recomendação do Conselho Regional de Serviço Social da 1ª Região nº 01/2020, de 20 de março de 2020^15^; 3. Resolução do Conselho Regional de Serviço Social de Sergipe (CRESS/SE) nº 01 de 25 de março de 2020^16^; 4. Nota Técnica nº 01/2020, emitida pelo Conselho Regional de Serviço Social do Acre^17^; 5. Resolução nº 007/2020 emitida pelo Conselho Regional de Odontologia de Minas Gerais^18^; 6. Resolução nº02/2019, de 16 de janeiro de 2019, emitida pelo Conselho Regional de Psicologia – Distrito Federal^19^; 7. Protocolo do Conselho Regional de Psicologia para o “Plano de Convivência da Secretaria Estadual de Saúde de Pernambuco para Enfrentamento da Pandemia do Coronavírus – covid 19”, publicado em junho de 2020^20^; 8. Resolução do Conselho Regional de Psicologia 15, nº 003, de 28 de novembro de 2019^21^; 9. Resolução do Conselho Regional de Psicologia - 18/MT nº002/2019 de 30 de janeiro de 2018^22^; 10. Resolução nº 01, de 11 de janeiro de 2019, emitida pelo Conselho Federal de Psicologia^23^; 11. Instrução Normativa nº 65, de 30 de julho de 2020, emitido pelo Ministério da Economia^24^; 12. Resolução do Conselho Federal de Medicina nº 2.235/2019 de 1 de outubro de 2019^25^; 13. Resolução do Conselho Regional de Medicina do Estado da Bahia nº 367/2020^26^.

Embora tais normas apresentem em seu conteúdo dispositivos que regulam ergonomia e ambiente de trabalho, elas pouco dizem acerca dos deveres do empregador para garantir que isso ocorra. Às vezes o sentido é até inverso, por exemplo, o conteúdo sobre condições de trabalho que consta do artigo 23 da Instrução Normativa do Ministério da Economia n. 65, de 30 de julho de 2020^24^:

Quando estiver em teletrabalho, caberá ao participante providenciar as estruturas física e tecnológica necessárias, mediante a utilização de equipamentos e mobiliários adequados e ergonômicos, assumindo, inclusive, os custos referentes à conexão à internet, à energia elétrica e ao telefone, entre outras despesas decorrentes do exercício de suas atribuições.

Embora no Brasil haja pouca preocupação com os riscos oriundos do teletrabalho, é necessário lembrar que, se há um processo de implantação de teletrabalho, o empregador deverá considerar todas as estratégias que possam diminuir ou controlar os fatores de riscos ocupacionais que podem afetar a saúde do trabalhador^27^. Nesse sentido, por exemplo, o ABC do Teletrabalho, na Colômbia, sobre a gestão de riscos ocupacionais no teletrabalho apresenta uma lista de verificação que inclui, entre outros aspectos, “a verificação de empregos, além de propor e implementar medidas de intervenção associadas ao local de trabalho e conscientizar sobre o autorrelato das condições de trabalho”^28^ (p. 96).

Entre os conselhos e recomendações dadas para a implantação do teletrabalho, no citado documento, estão:

filiação ao sistema integral de previdência social, conhecer as condições do local de trabalho do teletrabalhador, garantir que o teletrabalhador autorrelate suas condições de trabalho, acrescentando no Regimento Interno do Trabalho, a modalidade de contratação de teletrabalho, definir as horas estatutárias de trabalho por dia e por semana, fazer as correções necessárias para os ambientes de trabalho, processos de treinamento, treinamentos e treinamentos, entre outros^28^ (p. 99).

Por outro lado, o acordo acerca do teletrabalho entre agentes sociais na União Europeia, em 2002, “aponta a dificuldade de regulamentar essa nova forma de organização do trabalho e da prevenção de possíveis lesões e doenças associadas, especialmente transtornos mentais e distúrbios”^6^ (p. 1).

Os resultados encontrados atestam o crescimento rápido e exponencial do teletrabalho em saúde no Brasil, refletido na regulação normativa editada por diferentes órgãos e instituições reguladoras do país. Se até antes da pandemia o uso e a regulação do teletrabalho eram tímidos e avançavam de maneira gradual, a pandemia tratou de acelerar esse processo de institucionalização do teletrabalho em saúde no país, em um movimento que parece sem volta. Destaque-se, nesse sentido, o grande número de normas jurídicas publicadas nos anos de 2020 e 2021^9^, anos mais pesados da pandemia da covid-19, normas essas que mantém sua vigência mesmo após o final da emergência sanitária.

No entanto, ao mesmo tempo em que se aumentou o uso e a regulação dessa prática, não se verifica na regulação estatal editada uma proteção adequada seja do profissional de saúde, seja do paciente. Temas relevantes como os da jornada de trabalho, controle do tempo de trabalho, ergonomia e ambiente de trabalho foram completamente ignorados ou tratados de forma insuficiente, deixando trabalhadores e pacientes em situação de vulnerabilidade.

A compreensão do fenômeno do teletrabalho não pode ser dissociada dos movimentos do mundo do trabalho em geral, a respeito dos quais a literatura^29^ há muito apresenta reflexões críticas acerca das precárias condições do trabalho e dos seus efeitos deletérios à saúde do trabalhador, por exemplo, a Síndrome de Burnout, um tipo de estresse ocupacional que acomete profissionais envolvidos em qualquer tipo de cuidado em uma relação de atenção direta, contínua e altamente emocional^31^.

Uma pesquisa realizada sobre as condições ergonômicas em que os trabalhadores de diversos setores econômicos do Brasil exerceram o trabalho remoto no primeiro ano da pandemia causada pela covid-19 identificou que grande parte dos trabalhadores avalia essas condições como “razoáveis”, por disporem de alguns equipamentos tecnológicos, ou “péssimas”, por não terem espaço e condições para exercer o trabalho^32^_._ Observa-se que as avaliações se concentraram entre razoável e péssima, o que torna evidente a necessidade de acompanhar os movimentos de instituição de teletrabalho em saúde no Brasil e de dedicar especial atenção para a edição de normas jurídicas negligentes ou até deletérias no que se refere à proteção dos direitos do teletrabalhador na área da saúde no Brasil.

## CONSIDERAÇÕES FINAIS

A análise das normas jurídicas selecionadas permite identificar a ausência de normas editadas pelos órgãos e instituições reguladoras do trabalho em saúde no Brasil aptas a defender condições dignas de trabalho no campo do teletrabalho em saúde, e insuficientes para induzir empregadores a criarem condições favoráveis à realização do teletrabalho, seja ele realizado nos domicílios, seja em outro espaço privado. Essa é uma discussão necessária, relevante e complexa, que envolve direitos fundamentais, tais como, o sigilo e privacidade de dados pessoais; os deveres éticos; as responsabilidades dos profissionais de saúde; a proteção da saúde; e as condições de trabalho do empregado e do prestador de serviços. Por isso, os trabalhadores e gestores da saúde devem se manter atentos e agir rápido para proteger o trabalho, o emprego, o serviço e, sobretudo, o usuário/paciente.

Estudar o teletrabalho e seus efeitos no emprego, na prestação dos serviços de saúde e na integridade do trabalhador deve se constituir uma tarefa urgente para aqueles que atuam na área da gestão e da regulação do trabalho em saúde. Incluir esse tema na agenda do gestor poderá minimizar os possíveis efeitos deletérios para a gestão do trabalho em saúde e melhorar os conteúdos e as respectivas aplicações das normativas legais. Destaca-se a importância de se constar a saúde e a segurança do trabalhador como um dos elementos centrais nessa discussão.

Há muito o que se aprender sobre essa nova modalidade de trabalho, pois, conforme os resultados aqui apresentados, é possível que a realidade do teletrabalho seja incrementada após a pandemia. Dessa forma, reforça-se a necessária vigilância para que essa nova modalidade de trabalho em saúde e as tecnologias que lhe dão sustentação não aumentem as desigualdades já existentes no mundo do trabalho, nem contribuam para que os trabalhadores e usuários realizem suas atividades em ambientes inseguros.

A pesquisa realizada evidencia uma lacuna normativa a ser compreendida e superada, bem como desnuda o tratamento ainda primário, fragmentado e titubeante do Estado brasileiro para a regulação do teletrabalho em saúde em seus diversos aspectos. Nesse sentido, pretende-se chamar a atenção para a importância de se aperfeiçoar a regulação do teletrabalho, no sentido de que essa regulamentação, ao contrário da realidade atual, seja efetivamente protetora do direito à saúde dos pacientes e dos direitos do profissional da área da saúde.

Considerando que o teletrabalho já está presente nos sistemas público e privado de saúde, a sua agenda regulatória é um desafio urgente, e não do futuro, uma vez que essa realidade já faz parte do cotidiano das instituições de saúde.
